# Complete genome sequence of *Yokenella regensburgei* isolated from a patient with urinary tract infection in India

**DOI:** 10.1128/mra.01162-23

**Published:** 2024-04-30

**Authors:** Rani Diana Sahni, Aravind V, Thangamani Suji, Annie Sheeba V, Selvin Theodore Jayanth

**Affiliations:** 1 Department of Clinical Microbiology, Christian Medical College, Vellore, Tamil Nadu, India; 2 Department of Urology, Christian Medical College, Vellore, Tamil Nadu, India; Loyola University Chicago, Chicago, Illinois, USA

**Keywords:** *Yokenella*, WGS, AmpC, UTI

## Abstract

*Yokenella regensburgei*, an environmental organism, is an emerging pathogen in patients chiefly with immune suppression. We report the draft genome of *Y. regensburgei*, strain UU2206353, isolated from the urinary tract of an immunocompetent individual. The assembled genome consisted of 4,669,536 bp distributed over 20 contigs with 4,283 protein-coding genes.

## ANNOUNCEMENT

*Yokenella regensburgei* belongs to Order *Enterobacterales* (1). Multi-niche adaptation is evidenced by isolation from insects, reptiles, and well-water. Human skin/soft tissue infections, osteomyelitis, and sepsis, are described in immune-suppressed individuals (2–4).

We isolated *Y. regensburgei* from an immune-competent 69-year-old gentleman with benign prostatic hypertrophy and symptomatic lower urinary tract infection. Growth of >100,000 CFU/mL was obtained from a midstream urine and suprapubic aspirate (UU2206353). The isolated colonies on 7% sheep blood agar (SBA), after 37°C overnight incubation, were identified as *Y. regensburgei* by bioMérieux Matix-assisted Laser Desorption Ionization-Time of Flight- Mass Spectrophtometry (MALDI-TOF-MS). The Clinical and Laboratory Standards Institute methodology (CLSI-M100, Ed32) determined resistance to penicillin, aminopenicillins, cefoxitin, and colistin. Disk approximation test detected chromosomal *amp*C-mediated inducible cephalosporin resistance while family-specific *amp*C primers (5) failed to detect gene carriage. We employed Whole genome sequencing (WGS) to efficiently determine resistance cum virulence genes, enabling infection of the urinary system in an immunocompetent host.

Genomic DNA was extracted using QIAamp DNA Mini Kit (QIAGEN, Germany) from 15 isolated colonies on SBA after 37°C overnight incubation. Sequencing-ready, paired-end library was prepared using 100ng of DNA with the Nextera DNA sample preparation kit (Illumina Inc., San Diego, USA). Paired-end (2 × 150 bp) sequencing was performed on the Illumina NextSeq 500 system. The quality of paired-end reads was evaluated using fastQC (v0.11.9) (6) and assembled using skesa (v2.5.1) (7). Gene prediction was done using the Prokaryotic Genome Annotation Pipeline (PGAP) (v6.3) (8). Virulence and antimicrobial resistance (AMR) determinants were screened with abricate (v1.0.1) using Virulence Factor Database (VFDB) (9) database and NCBI database (v3.10) (10), respectively.

For comparison, *Y. regensburgei* (*n* = 11) publicly available assembled genomes were retrieved from NCBI (https://www.ncbi.nlm.nih.gov; 21 January 2023): human wound (1), gastrointestinal tract (2), insects (3-Eastern boxelder, *Pyrrhocoris apterus*), environment (2-sewage, urban dwelling), and unknown sources (3).

The “gff” files generated after annotation by bakta (v1.6.1|DB: 4.0.0) (11) were subjected to pangenome analysis using Panaroo (v1.3.2) (12). Core genome alignment was used for constructing phylogenetic tree by Fasttree (v2.1.11) (13) according to the GTR-GAMMA model. Genomic clusters were defined by Rhierbaps (v1.1.4). The phylogenetic tree was annotated by iTOL (https://itol.embl.de) (14). The default parameters were used to run all tools on assembled genomes.

The assembled genome consisted of 4,669,536 bp distributed over 20 contigs. It contained 4,424 protein-coding sequences, 77 tRNAs, and 4 rRNAs. This isolate was identified as *Y. regensburgei* by Average Nucleotide Identity analysis by NCBI and showed 99.32% similarity to *Y. regensburgei* strain NCTC11966 (GCA_900460805.1). The genes predicted by VFDB and NCBI database are provided in (Table 1). Gene prediction after functional annotation showed a unique *bla*_YOC-1_ (class C beta-lactamase) (15) with *amp*E (beta-lactamase regulator), beta-lactamase superfamily protein and MBL fold metallo-hydrolase genes.

**TABLE 1:** Virulence gene carriage of *Yokenella regensburgei* UU2206353

Sample	Contig	Start	End	Gene	%Coverage	%Identity	Database	Accession	Product
UU2206353	3	785673	787847	iroN	100	80.83	vfdb	WP_015874979	(iroN) salmochelin receptor IroN [Sal (VF0563) - Nutritional/Metabolic factor (VFC0272)] (*Klebsiella pneumoniae* subsp. pneumoniae NTUH-K2044)
UU2206353	3	834117	835283	ugd	100	81.23	vfdb	WP_004175261	(ugd) UDP-glucose 6-dehydrogenase [Capsule (VF0560) - Immune modulation (VFC0258)] (*Klebsiella pneumoniae* subsp. pneumoniae NTUH-K2044)
UU2206353	3	835522	836928	gndA	100	83.01	vfdb	WP_014907233	(gndA) NADP-dependent phosphogluconate dehydrogenase [Capsule (VF0560) - Immune modulation (VFC0258)] (*Klebsiella pneumoniae* subsp. pneumoniae NTUH-K2044)
UU2206353	3	847069	847955	galF	99.55	80.72	vfdb	WP_001741945	(galF) GalU regulator GalF [Capsule (VF0560) - Immune modulation (VFC0258)] (*Klebsiella pneumoniae* subsp. pneumoniae NTUH-K2044)
UU2206353	3	1010038	1010688	rcsB	100	88.33	vfdb	WP_002913007	(rcsB) transcriptional regulator RcsB [RcsAB (VF0571) - Regulation (VFC0301)] (*Klebsiella pneumoniae* subsp. pneumoniae NTUH-K2044)
UU2206353	3	1164811	1165302	hcp/tssD	100	85.98	vfdb	WP_002902160	(hcp/tssD) type VI secretion system protein Hcp family [T6SS (VF0569) - Effector delivery system (VFC0086)] (*Klebsiella pneumoniae* subsp. pneumoniae NTUH- K2044)
UU2206353	3	778441	779553	iroB	100	87.87	ncbi	EOW04219.1	Salmochelin biosynthesis C-glycosyltransferase IroB
UU2206353	3	779628	783278	iroC	99.67	81.87	ncbi	AUH19662.1	Salmochelin/enterobactin export ABC transporter IroC
UU2206353	9	531830	532987	blaYOC-1	100	99.57	ncbi	MT271603.1	*Yokenella regensburgei* blaYOC
UU2206353	14	190476	191467	rpoS	99.9	89.52	vfdb	NP_461845	(rpoS) RNA polymerase sigma factor RpoS [RpoS (VF0112) - Regulation (VFC0301)] (*Salmonella enterica* subsp. enterica serovar Typhimurium str. LT2)
UU2206353	20	50975	51871	fieF	99.67	88.63	ncbi	BAB89353.1	CDF family cation-efflux transporter FieF
UU2206353	21	219196	222338	acrB	99.49	83.22	vfdb	WP_002892069	(acrB) acriflavine resistance protein B [AcrAB (VF0568) - Antimicrobial activity/Competitive advantage (VFC0325)] (*Klebsiella pneumoniae* subsp. pneumoniae NTUH-K2044)
UU2206353	21	222361	223554	acrA	99.75	80.53	vfdb	WP_004177236	(acrA) acriflavine resistance protein A [AcrAB (VF0568) - Antimicrobial activity/Competitive advantage (VFC0325)] (*Klebsiella pneumoniae* subsp. pneumoniae NTUH-K2044)
UU2206353	21	337142	339349	fepA	98.25	82.06	vfdb	WP_012737617	(fepA) outer membrane receptor FepA [Ent (VF0562) - Nutritional/Metabolic factor (VFC0272)] (*Klebsiella pneumoniae* subsp. pneumoniae NTUH-K2044)
UU2206353	21	344838	345609	fepC	97.11	81.35	vfdb	WP_002893737	(fepC) iron-enterobactin transporter ATP-binding protein [Ent (VF0562) - Nutritional/Metabolic factor (VFC0272)] (*Klebsiella pneumoniae* subsp. pneumoniae NTUH-K2044)
UU2206353	21	345622	346504	fepG	88.92	80.29	vfdb	WP_004147521	(fepG) iron-enterobactin transporter permease [Ent (VF0562) - Nutritional/Metabolic factor (VFC0272)] (*Klebsiella pneumoniae* subsp. pneumoniae NTUH-K2044)
UU2206353	21	347729	348946	entS	97.99	80.31	vfdb	WP_004147525	(entS) enterobactin exporter EntS [Ent (VF0562) - Nutritional/Metabolic factor (VFC0272)] (*Klebsiella pneumoniae* subsp. pneumoniae NTUH-K2044)
UU2206353	21	352935	353779	entB	99.18	83.67	vfdb	WP_004210873	(entB) 23-dihydro-23-dihydroxybenzoate synthetase isochorismatase [Ent (VF0562) - Nutritional/Metabolic factor (VFC0272)] (*Klebsiella pneumoniae* subsp. pneumoniae NTUH-K2044)
UU2206353	21	353798	354540	entA	99.2	82.55	vfdb	WP_086074289	(entA) 23-dihydroxybenzoate-23-dehydrogenase [Ent (VF0562) - Nutritional/Metabolic factor (VFC0272)] (*Klebsiella pneumoniae* subsp. pneumoniae NTUH-K2044)
UU2206353	21	415262	415698	fur	96.47	83.98	vfdb	NP_459678	(fur) ferric iron uptake transcriptional regulator [Fur (VF0113) - Regulation (VFC0301)] (*Salmonella enterica* subsp. enterica serovar Typhimurium str. LT2)
UU2206353	21	714780	715832	ompA	99.71	86.08	vfdb	AAF37887	(ompA) outer membrane protein A [OmpA (VF0236) - Invasion (VFC0083)] (*Escherichia coli* O18:K1:H7 str. RS218)

**Fig 1:**
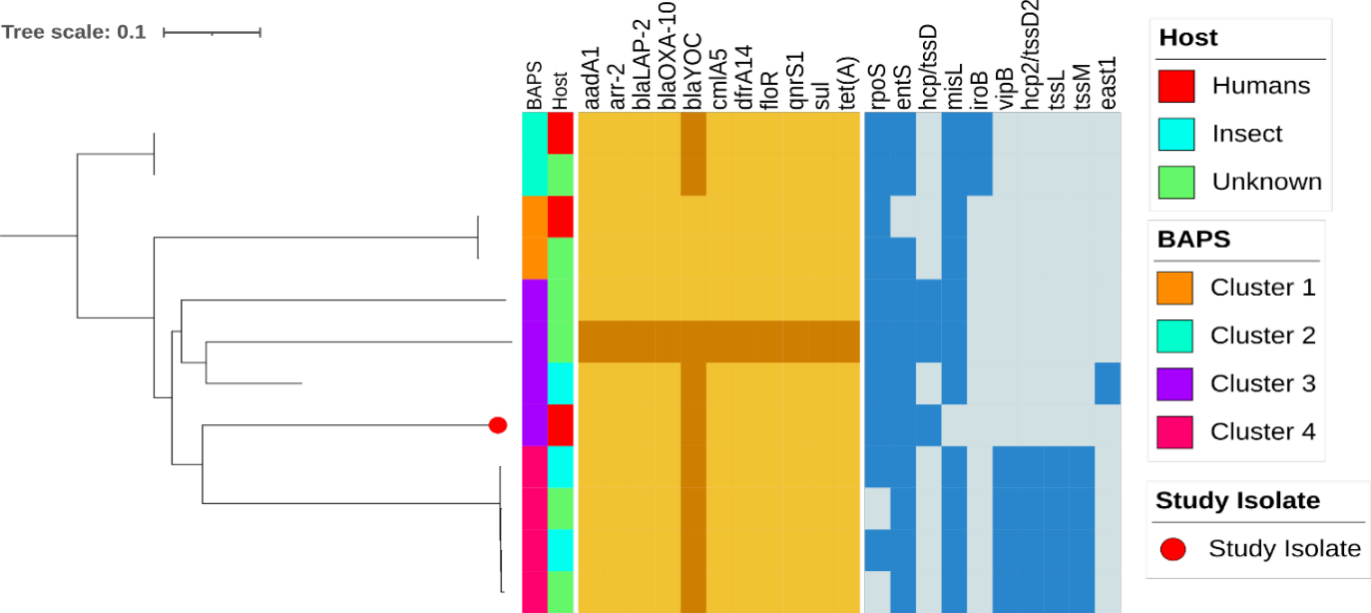
Core genome phylogenetic tree. Phylogenetic analysis of *Y. regensburgei* based on core genome phylogenetic tree. Maximum-likelihood phylogenetic tree constructed with GTR-GAMMA model using Fasttree and rooted with ATCC-43003 strain (Accession No: GCA_000239335.1). The position of study isolate is marked by a red dot at the branch tip. Genomes with their associated metadata are labeled as color strips. Each isolate’s BAPS cluster and Host are shown in color strips 1 and 2, respectively. Pirate Gold color heatmap represents the AMR genes of each isolate in study. Blue color heatmap represents the virulence gene distributions. Color keys for all the variables are given in the inset legend. The tree was visualized and labeled using iTOL (https://itol.embl.de/).

The core genome phylogenetic tree rooted with GCA_000239335.1 (ATCC-43003 strain) revealed four primary BAPS clusters. The study isolate belongs to cluster 3 (Fig. 1). No host specificity was identified in the core genome tree. Distribution of AMR and virulence genes also showed little correlation within the same core genome clade. Pan-genome analysis showed 3,727 core genes (60.6%, 3,727/6,151) and highly strain-specific accessory genes (2,424/6,151). Finally, in this emerging pathogen, we did not find notable genomic differences indicating niche adaptation.

## Data Availability

The whole-genome assembly has been deposited at GenBank under accession number GCF_030867855.1. Raw reads were deposited under SRA accession number SRR23190116 with BioSample accession number SAMN32873490.
